# High-Frequency Excitation and Surface Temperature Analysis of Breast Tissue for Detection of Anomaly

**DOI:** 10.1155/2023/4406235

**Published:** 2023-02-08

**Authors:** Benjamin Jessie, Jingchen Liang, Yinan Li, Babak Fahimi

**Affiliations:** ^1^Department of Electrical Engineering, University of Texas at Dallas, Richardson, TX, USA; ^2^Ansys Inc., San Jose, CA, USA

## Abstract

Techniques used for breast cancer detection usually incorporate Infrared Thermography (IRT) to locate abnormal hotspots or asymmetry in a thermal texture map. This can be unreliable due to various individual differences from one person to another. In this paper, a detection method that is independent of the aforementioned limitations is proposed. This technique is a combination of thermal imaging and high-frequency excitation. This technique is based on the fact that the differences in electromagnetic and thermal properties of abnormal (malignant) tissue and the surrounding normal tissue will result in a noticeable difference in temperature increase after exposure to high-frequency excitation. A three-dimensional (3-D) finite-element method (FEM) has been used to simulate the thermal behavior of breast tissue exposed to antenna excitations. Finally, the effectiveness of this technique was tested in a series of experiments using a life-sized breast phantom.

## 1. Introduction

Breast cancer is the second leading cause of cancer death in women [1]. Early detection of cancerous tissue is the key to increasing the survival rate. Techniques for breast cancer detection have been studied by many scholars for decades, and they are mainly classified into two categories based on whether or not the diagnostic equipment is in contact with patients [2]. The first, invasive methods, includes mammography and biopsy. These procedures require extracting tissue samples or close contact between the test equipment and the human body. The second category of noninvasive methods include Infrared Thermography (IRT), Ultrasonic Imaging, Microwave Radiometry (MR), Magnetic Resonance Imaging (MRI), and Electrical Impedance Tomography (EIT) [2].

Unfortunately, the traditional techniques mentioned above usually suffer from discomfort, risks of overexposure to radiation, unreliability, and high cost. In this paper, a novel, nonintrusive method using high-frequency antenna excitation in combination with thermal imaging has been proposed. The goal is to satisfy the ideal breast cancer detection criteria of safety, comfort, high accuracy, and affordability.

Different from the traditional IRT method, which depends on thermal graphs of healthy breast models to help determine the existence of an abnormal pattern, the proposed method is based on the unique thermal and electromagnetic properties of tumorous tissues. Mini-patch antennas were utilized to serve as a source of electromagnetic excitation for the breast. The temperature data was obtained on the skin's surface directly under the antenna using nonintrusive thermal sensors. This method magnifies the abnormal temperature shown on the skin's surface due to the presence of malignant tissue. It also superimposes thermal data captured from the entire breast, thereby achieving a highly reliable and accurate thermogram [3].

## 2. Methods and Modeling

### 2.1. Methods

Detection of breast cancer is essentially to determine the existence and the location of the tumor. Breast cancer most often begins with cells in milk-producing ducts and glandular tissue [4]. Breast tumors are a type of abnormal and excessive growth of tissue that eventually forms a mass. The abnormal growth of tissue is due to the growth of abnormal cells, which lose their ability to stop dividing and dying. In its early stages, the tumor appears like a lump that feels different from the rest of the breast tissue. This stage is called the “in situ” stage, which means that the cancerous cells stay within their original tissue compartment and have not spread to the surrounding tissues. It is the stage when treatment is most effective. As more cancer cells develop, they invade into other parts of the breast or body. This is referred to as “invasive” breast cancer. Cancer treatment at this stage is extremely difficult [5].

Cancerous tissue in breasts, like most other cancerous tissues, exhibits higher metabolic activity when compared to normal tissues surrounding them. This is due to their rapid pace of cell growth [6]. These differences in energy consumption lead to small but detectable temperature changes on the surface of the breast. This serves as the basis for breast cancer detection using thermal imaging. Although thermography has been studied for decades, the mechanisms of heat transfer between diseased and native tissue, and the differences between the tissues themselves, have not been well described. Moreover, this method assumes that each breast has a particular thermographic pattern that does not change over time. This suggests that one can take a baseline and mark any significant changes observed in later images for future analysis [7]. However, this assumption is not as reliable as once believed. Human breasts are fairly complex organs made of various tissues and a labyrinthine of blood vessels. The particular thermographic pattern could experience significant changes depending on the physical conditions of different individuals. Also, if the patient has an asymmetrical body temperature, analysis of the thermograms could result in even higher false-negative and false-positive rates [2].

In this paper, the proposed method is based on conventional thermal imaging, but is combined with external high-frequency excitation. The main advantage of this method over the conventional technique is the independence of the symmetry and particular thermographic patterns of a normal breast. Patch antennas were used to serve as a source of electromagnetic excitation to the breast. The radio frequency waves radiated by the antennas establish electric fields inside the entire breast including the tumor tissue. Because of the differences in the electromagnetic and thermal properties of tumorous and normal tissues, radio frequency excitation will cause the tumor to heat up more than the surrounding healthy tissue. This is due to the tumor resisting the rotation of its molecular dipoles when exposed to the external electric field. This increased resistance leads to an increase in losses, and that becomes evident through the increased amount of released heat. The differences in the thermal response of the healthy and diseased tissues can be measured from the surface of the breast, so the portion of the breast directly above the tumor will have a higher temperature than the surrounding area. However, since the mechanisms of heat transfer between diseased and native tissues and the differences between the two are not quantified, it is still not possible to uniquely detect the presence of a tumor. To make the recorded temperature data meaningful, this group of antennas will keep their relative position from each other while rotating a small angle along the circumferential edge of the breast. After each rotation, a new set of excitations (one from each antenna) is performed. In this way, the antenna group rotates and excites the breast multiple times to cover the entire surface of the breast. After each set of excitations and data recordings, the breast will be left to cool down to room temperature. A set of data containing the pre- and postexcitation surface temperatures will be collected. After analyzing the temperature trend, it can be determined if cancerous tissue is present, and its approximate location can be estimated.

### 2.2. Structure of Human Breast

In order to conduct research and develop a reliable method for breast cancer detection, having a firm understanding of the structure of human breasts is highly important.

**Figure 1:**
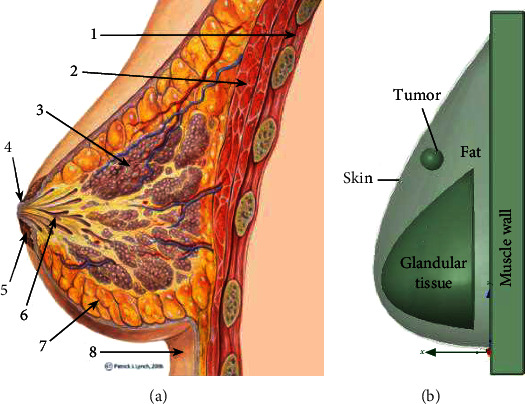
(a) Structure of human breast: (1) chest wall, (2) pectoralis muscles, (3) lobules, (4) nipple, (5) areola, (6) milk duct, (7) fatty tissue, and (8) skin. (b) cross-sectional view of FEA model of the human breast.

Figure 1(a) illustrates a cross-sectional view of a normal female human breast. It is a tear-shaped mammary gland that is designed to produce and secrete milk to feed infants. The breast consists of multiple layers of tissue with the majority of them being adipose (fat) and glandular tissue. Below the skin layer is the adipose tissue layer that extends all the way to the chest wall and spreads between the glandular tissues. The adipose layer envelops a network of milk ducts which are originated from the glandular tissue called lobules, where milk is produced and stored.

### 2.3. Multiphysics Modeling of the Detection System

Finite element analysis (FEA) is utilized to simulate the detection system for the proof of concept. The FEA is a numerical method for solving problems of engineering and mathematical physics, given the controlled parameters. FEA subdivides a large complex problem into smaller, simpler parts that are called finite elements. The analytical solution of these problems generally requires the solution to boundary value problems for complex partial differential equations, such as Maxwell Equations in this case. Since this detection system is a multidisciplinary-based system that involves electromagnetic analysis and heat transfer, as well as complex geometric model, analytical solutions would be extremely difficult to derive. Building a finite element model is the most efficient and accurate way to estimate the behavior of the system. In this case, modeling is through ANSYS HFSS and coupled with ANSYS Mechanical (Workbench).

The breast model was directly drawn in High-Frequency Simulation Software (HFSS). In order to have more accurate results, and make the model as close as possible to reality, the general shape of the breast was designed into the shape of a tear. This is unlike most other studies where a hemisphere is employed. This model consists of the four most important layers of the breast: skin, fat, gland, and muscle.  Figure 1(b) illustrates the 3-D breast model and its cross-sectional view depicting the distribution of different layers including a tumor. As can be seen from the figure, this model follows the general structure of the breast shown in Figure 1(a), where the glandular tissue in the center was surrounded by layers of adipose (fat). The outermost layer is skin, whose thickness varies. Last but not least, everything is sitting on the chest wall that is made of muscle. Other less important parts of the breast, such as the nipple and areola, can be reasonably neglected. Since this method is designed for tumor detection, a malignant tumor was also modeled. The frequency-dependent electromagnetic properties of each layer was included in this model. A patch antenna was chosen to be the external excitation source since they usually have a low profile and can be mounted on a flat surface.

**Figure 2:**
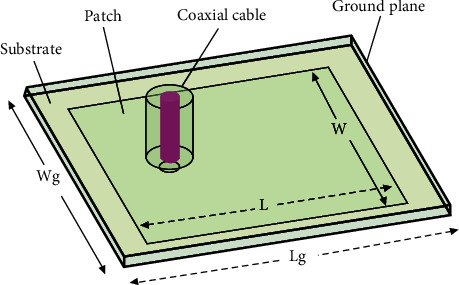
Probe feed patch antenna.

Patch antennas are also very practical at microwave frequencies. Because wavelengths are so short at these frequencies, the patches can be made conveniently small enough to be mounted near the surface of human breasts. Generally, a basic patch antenna consists of a patch sitting on top of a dielectric substrate and a ground plane.  Figure 2 demonstrates the patch antenna that was modeled in HFSS. The distance between the patch and the ground plane equals the height of the substrate, which determines the bandwidth. The resonant frequency of the antenna is determined by the length and width of the patch as well as the ground plane. The length and width of the patch and ground plane were calculated by the following equations where *L* and *W* are the length and width of the patch, respectively; *L*_*eff*_ is the effective length of the patch; *h* is the height of the dielectric substrate; *f* is the operating frequency; *ε*_*r*_ is the dielectric constant; and *ε*_*eff*_ is the effective dielectric constant. The values of the parameters are listed in Table 1 [8].

(1) $$\begin{array}{c} L=L_{eff}-2\Delta L,\\ \Delta L=0.412h\frac{\left(\varepsilon_{eff}+0.3\right)\left(W/h+0.264\right)}{\left(\varepsilon_{eff}-0.258\right)\left(W/h+0.8\right)}\\ L_{eff}=\frac{c}{2f\sqrt{\varepsilon_{eff}}},\\ \varepsilon_{eff}=\frac{\varepsilon_{r}+1}{2}+\frac{\varepsilon_{r}-1}{2}\left(\frac{1}{\sqrt{1+12h/W}}\right). \end{array}$$

### 2.4. System Integration

**Figure 3:**
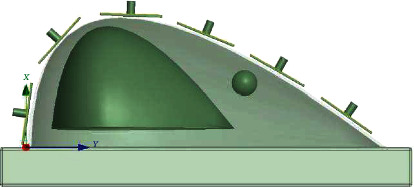
Patch antenna distribution on the breast surface.

**Figure 4:**
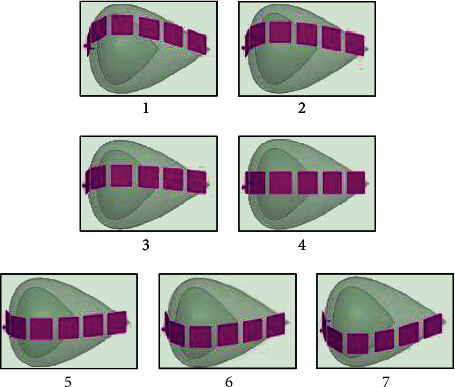
Rotation of antenna group to cover breast surface.

This method employs a group of antennas to excite the breast multiple times. The arrangement of the antenna groups is shown in Figure 3, where six antennas were used to cover the longitudinal edge of the breast. The entire surface of the breast will be covered by rotating the antenna lines along the transversal direction as shown in Figure 4.

Since temperature distribution is the key to this detection method, the electromagnetic simulation results were then coupled to ANSYS Mechanical to perform the thermal analysis. In this analysis, *ω*, *q*_*m*_, *ρ*_*b*_, and *c*_*b*_ are the blood perfusion rate, metabolic heat generation rate, blood density, and specific heat, respectively; the bioheat transfer process with Pennes' bioheat equation was employed [9]:

(2) $$\begin{array}{c} \frac{d^{2}}{{dx}^{2}}+\frac{q_{m}{\omega \rho }_{b}c_{b}\left(T_{a}-T\right)}{k}=0. \end{array}$$

**Figure 5:**
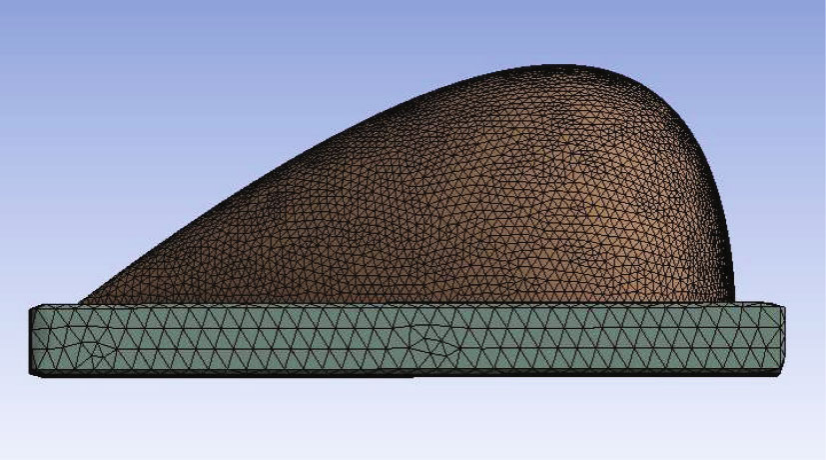
Mesh assignment of breast model.

Thermal properties of different tissues were predefined in the “Engineering Data” of the software and later assigned to their corresponding portion of the breast in the simulation interface.  Table 2 summarizes both electromagnetic and thermal properties of different tissue layers used in this analysis [10, 11]. Radiation and convection heat transfer boundary conditions were assigned to the surface of the skin, and a constant temperature boundary was applied to the muscle layer to represent body temperature. The ambient temperature is set the same as the body's internal temperature (37°C) in order to balance the metabolic heat generation and heat loss through blood perfusion. Therefore, any heat gain is a sole result of the external EM source, and heat loss is due to convection and radiation. To have accurate FEA results, proper mesh assignments are mandatory. Usually, a finer mesh will lead to more accurate results. However, extrafine meshing will result in an unnecessarily long computational time without always providing a significant difference in the results' accuracy. The most finely meshed areas of the model are the skin and tumor. This is because the skin layer is the thinnest part of the model, and the tumor has the smallest volume in the model. More mesh is required to allow more computational freedom. The mesh quality standard was determined by parameters such as orthogonal quality and skewness. Orthogonal quality is the measure of how close angles between mesh element edges and faces are to some optimal angle. It is measured on a scale of 0 to 1, with 1 being the best. Skewness is the geometrical orientation of a mesh. It is a measure of how much the generated mesh differs from the ideal mesh cell. It is measured on a scale of 0 to 1, with 0 being the best.  Figure 5 depicts the mesh assignment of the breast model. This simulation was run for 30 seconds to accommodate for the maximum allowed temperature and SAR tolerance of human tissue. The sensitivity of this method, with respect to excitation time, will be discussed later.

## 3. Multiphysics Simulation Results

In order to make the results more applicable to realistic clinical studies, the specific absorption rate (SAR) and maximum temperature increase were checked to ensure they were within safety regulation. SAR is a measure of the energy absorption rate by the human body when exposed to a radio frequency electromagnetic field.

**Figure 6:**
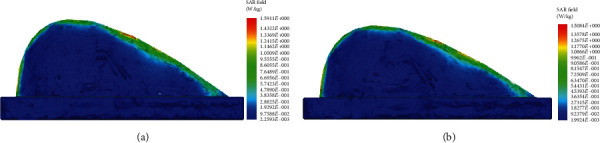
Local SAR field of the breast (a) with embedded tumor and (b) without tumor.

**Figure 7:**
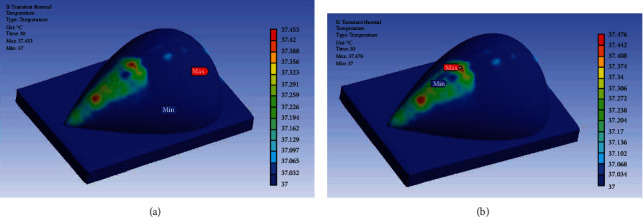
Temperature distribution on (a) breast model with embedded tumor and (b) healthy breast model.

According to the American national standard, the IEEE International Committee on Electromagnetic Safety requires that the SAR value averaged over the entire body may not exceed 4 W/kg when exposed to frequencies between 3 kHz and 300 GHz. This standard is set to ensure that individuals do not experience negative health effects due to electromagnetic radiation [9]. Additionally, 1.6 W/kg averaged over 10 g of tissue is the maximum allowed localized SAR.  Figure 6 illustrates the local SAR distribution over both the normal and cancerous breast models. Although there is a similar SAR distribution for both models, the legend of the cancerous breast model has a higher maximum SAR value, indicating more absorption. Even with the increase in SAR due to the presence of the embedded tumor, neither model exceeds the maximum value allowed. The maximum temperature increase in the human body should be less than 1°C according to the IEEE standard [12].  Figure 7 depicts the temperature distribution on a human breast with and without an embedded tumor after 30 seconds of excitation. Neither model exceeds the maximum temperature increase allowed. Again, although both simulations had similar temperature patterns, the maximum temperature in the legends varied.

Notice that in Figure 6(a), the maximum temperature label seems to be incorrect. This is due to the presence of the glandular tissue being near the surface of the skin causing a very localized maximum temperature. An antenna was not placed directly above the tumor during excitation, so it did not receive the same level of excitation as the other breast tissue directly under the antennas. The tumor's temperature increase was larger than that of the surrounding tissue, but not significant enough to cause a large thermal marker on the surface on the breast. In Figure 7(a), the minimum temperature is 37°C, so the minimum temperature label can be located at any position at the temperature. Although there is more than one location with that minimum temperature, the label is applied to one of the locations that is surrounded by excited tissue that did increase in temperature.

**Figure 8:**
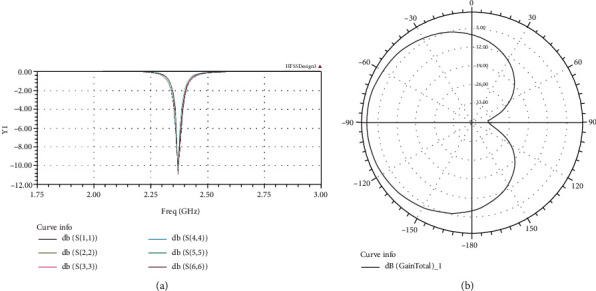
Group antenna return loss (a) and antenna radiation pattern (b).

It was important that all six antennas resonated at the designed frequency (2.4 GHz) and radiated the same amount of energy.  Figure 8(a) demonstrates the behavior of all six antennas, and it can be seen that the return losses were minimum at around 2.4 GHz.  Figure 8(b) depicts the radiation pattern of each antenna.

**Figure 9:**
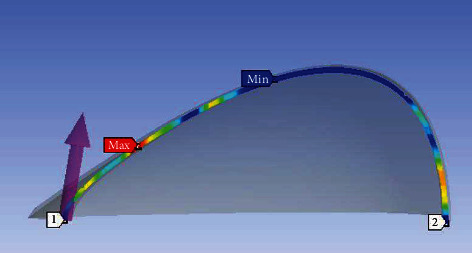
Simulated temperature measurement convention.

**Figure 10:**
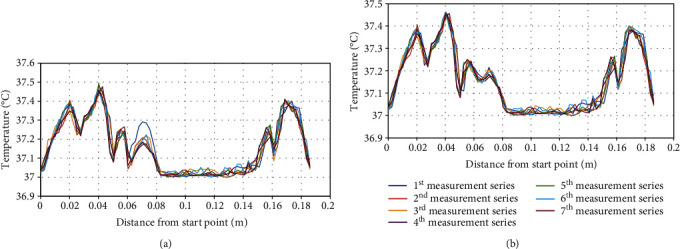
Temperature distribution along the circumferential line on (a) a breast with tumor and (b) a breast without tumor.

In the postprocessing procedure after each simulation, the temperature along a circumferential line located right below the rotating antennas on the skin surface was measured. The measurements' start and end points are shown in Figure 9. The tumor was modeled into a location in the breast fat tissue as shown in Figure 1(b). Seven simulations corresponding to seven different locations of the antenna group have been performed to plot the surface temperature trend. The results are plotted in Figure 10(a) and also compared to the breast without the embedded tumor in Figure 10(b). The horizontal axis is the distance from the measurement starting point, while the vertical axis is the temperature. Seven measurement series correspond to seven excitations with different antenna group locations. Several temperature hotspots were shown in the plot corresponding to each antenna location. It can be seen that the temperature distribution along the antenna line before and after rotation has a similar pattern, with similar peaks and troughs except at one location: at approximately 0.07 m. Based on Figure 1(b), this is approximately where the tumor is located. The reason for this difference in temperature is due to the sudden change of material properties as the antennas rotate. In areas where there is no tumor embedded, the tissue properties are consistent. As the antenna rotates, the established electric field in the breast is approximately the same, thus resulting in the same temperature rise, while in an area where a tumor is embedded, the EM waves experience a sudden change in material properties between two consecutive excitations. This causes a difference in the induced electric field and results in a different temperature increase. Different from conventional thermography, the use of an external excitation source reveals the changing trend of surface temperatures between healthy and malignant areas, so this method does not require comparison between particular thermographs.

## 4. Experimental Setup and Verification

**Figure 11:**
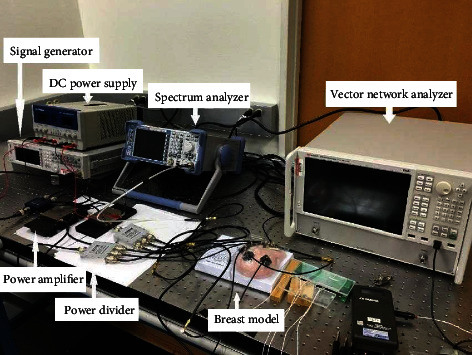
Experimental setup.

In previous sections, the multiphysics simulation process of a novel breast cancer detection technique based on the combination of high-frequency excitation and thermal analysis has been addressed in detail. In this section, the aforementioned FEA model and the detection technique will be tested on a prototype, and the experimental measurements will also be compared to the simulation results. The detection system setup is shown in Figure 11. This system consists of a high-frequency signal generator, a DC power supply, a spectrum analyzer, two power amplifiers, three power dividers, a vector network analyzer, several thermistors, several patch antennas, and a digital thermometer. Information about the equipment used can be found in the Appendix (available here).

This system starts from the analog signal generator which was used to generate the RF signal at the desired frequency. It has a frequency range from 9 kHz to 3 GHz. The DC power supply was used to power up the power amplifiers. Since the output power of the signal generator was only up to 19 dBm (less than 80 mW), it is necessary to use the power amplifiers to boost the output signals. An individual two-way power divider was employed before the two power amplifiers to split the output signal from the signal generator into two output signals of equal magnitude. These two output signals were used as inputs to the two amplifiers. The boosted output signals from the two power amplifiers were used as inputs to the two three-way power dividers to further split the power into six output signals with the same magnitude. The spectrum analyzer measures the magnitude of the output signals of each output port of the 3-way dividers versus frequency of the signal generator.

**Figure 12:**
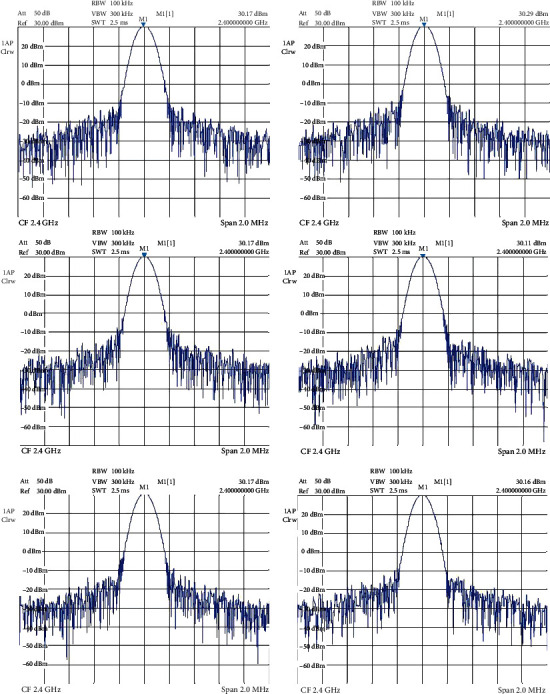
Output powers from each output port of the two 3-way power dividers.

**Figure 13:**
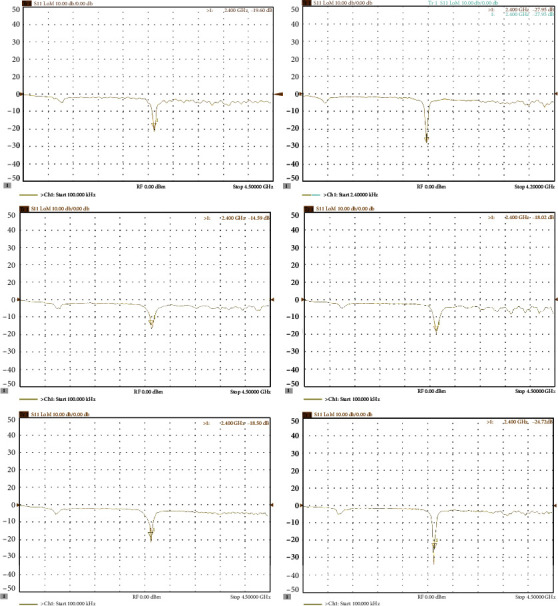
Return loss of patch antennas.

As can be seen from Figure 12, the final output powers from each port of the two 3-way power dividers are approximately the same. They were all measured as 30 dBm (1 W). The vector network analyzer is used to measure the network parameters of an electrical network. In this study, it was used to evaluate the performance of the patch antennas. As can be seen in Figure 13, all six patch antennas have a very low (if not the lowest) power reflections at 2.4 GHz. Most of the reflected power is lower than -20 dB, which means only 1% of power is reflected back from the antennas.

**Figure 14:**
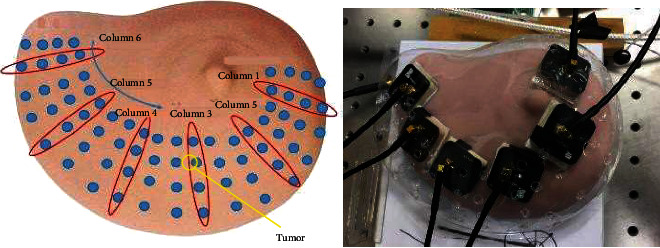
Patch antenna distribution on the breast model.

Figure 14 demonstrates the patch antenna distribution on the breast model. The tumor location is indicated by the yellow circle. The plastic shell was used to hold the antennas. It also helps in fixing the relative locations between the antennas and the breast, as well as maintaining equal distance between each antenna. The solid blue circles stand for the holes on the plastic shell. The antennas were arranged in such ways that their sides were parallel to each other. On the plastic shell, there were four rows of holes shown in the red circles (the rows are along the direction of the arrow in Figure 14) covering the majority area of the breast surface along the radial direction. During the first round of the experiment, all the antennas were fixed into the first row of holes and excited using the signal generator. After the temperature under each antenna is measured, the entire antenna group will be moved to the next row. In this way, four sets of excitations were performed by the antenna group. In addition to the antenna group being excited from each row, they were also moved from one column to another in the tangential direction. Because only six antennas were used, each row has an additional twelve unused columns to allow for the group to be shifted in the tangential direction of the breast. As a result, a complete test contains 72 holes and 12 sets of excitations in total. Both initial and final temperatures were measured. Between excitation periods, the model was allowed to cool to room temperature. Although the room temperature fluctuates, the excitation period is so short (20-45 s) that the change in room temperature can be reasonably disregarded.

**Figure 15:**
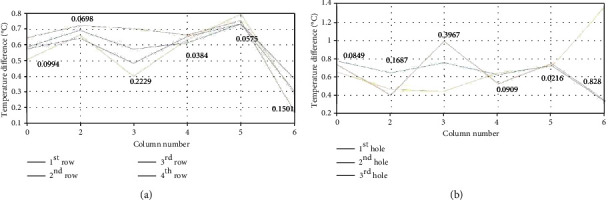
Temperature difference for (a) different rows and columns and (b) different holes and columns in the second row.

The experiment was first performed by placing the antennas in the location indicated by the 24 red circles. The excitation lasted for 30 s. Their initial and final temperatures were measured and compared. As can be seen in Figure 15(a), although the initial temperature is different for each point, the induced temperature increase in a given column is approximately the same for most cases except in column 3 at row 2, where the temperature increase is noticeably higher than the surrounding points. This difference in temperature increase is due to the presence of an abnormal tissue, in which the established electric field is different from those in the surroundings areas. As shown in Figure 14, each row contains two additional holes, for every column circled in red, to place antennas. This was done to allow the antennas to be moved along the tangential direction of the breast. Based on the antenna placements from Figure 14 and while remaining in the same row, each antenna can be moved one position to the right in the tangential direction to represent the first hole of the given column number, remain in the current position to represent the second hole of the given column number, or move one position to the left in the tangential direction to represent the third hole for the given column number. In the second test, the antenna group was moved to the second row, and the antennas were moved along the tangential direction on the breast surface. The excitation time was also 30 s.  Figure 15(b) illustrates the temperature difference between the initial and final temperatures for different holes along the second row at different columns. This figure shows a fairly constant temperature increase among different columns, but the variations within the same column is larger, especially for columns 3 and 6. The larger variation among different holes in column 3 is consistent with simulation results. The tumor location is between the second and third holes in column 3. This resulted in a larger variation than the temperature difference between the first and second holes of column 3 and the first and third holes of column 3, thus exposing the location of the tumor because of the increased temperature. The large variation in the sixth column is due to the existence of a fibrocystic mass in the corresponding location in the breast model.

## 5. Comparison of Simulation and Experimental Results

Results from the simulated data and experimental results suggest that the presence of a tumor does cause an elevated surface temperature of the breast after exposure to high-frequency excitation. Figures 10(a) and 10(b) display the temperature distribution along the circumferential line of the simulated breast model with and without a tumor, respectively. In Figure 10(a), it can be seen that during measurement 6 series, there is a noticeably larger increase in temperature at 0.07 m when compared with other measurement series at the same distance. This is the only position with a relatively drastic increase in temperature across all measurements and distances, and it is directly above the tumor. The increase in temperature was appropriately 0.29°C. In Figure 10(b), where there was no tumor present in the breast model, that difference in temperature increase did not occur. The temperature changes relative to position remained consistent for all 7 measurement series.

In the experimental results, a similar trend was followed with the area directly over the tumor having a slightly elevated temperature when compared with the surrounding area. This can be seen in Figures 15(a) and 15(b). Figure 14 shows that the tumor in the breast phantom is located in the second row between the second and third holes of column 3. Figure 15(a) shows that the largest difference in temperature increase occurred in row 2 of column 3, where the tumors is located. Column 3 exhibits the largest standard deviation in temperature increases, indicating that the variation in the increased temperature was larger than in any other column. Within column 3, row 2 had the largest increase in temperature, confirming the tumor's location as indicated in Figure 14. At this position, the temperature increased around 0.7°C.

Although over two times larger than the simulated temperature increase, the trend of increased temperature directly above the tumor remains consistent. In Figure 15(b), the surface temperature increases are displayed as a result of moving the antennas in the tangential direction for excitation. Each antenna was placed in row 2, and the temperature increase was measured as a result of the excitation from the first, second, and third holes of each column. While excluding column 6 due to the fibrocystic mass, it can again be seen that the three holes from column 3 have a significantly larger variation in temperature than the remaining columns. Hole 2 has the largest temperature increase, nearly 1°C, again confirming the location of the tumor with even more accuracy. This temperature increase is over three times larger than the simulated increase, but also follows the trend that the area directly above the tumor will have a larger temperature increase than the surrounding areas.

The differences in the temperature increase from the two experiments are due to the location of the thermal measurement. In the second experiment, where the row remained the same and the measurements were taken from different holes in each column, the temperature was measured closer to the actual location of the tumor resulting in a larger temperature increase. The difference in the simulated and experimental temperature increases are caused by the inconsistent environment during the experiment.

The simulation began with a set ambient temperature, so the only heat generated was due to excitation, and the cooling effects allowed the minimum temperature of the system to only be 37°C. After each simulation, the antennas were placed in a different position, and the system returned to the same initial conditions. This allowed for the final temperatures to be dependent only on the excitation from the antennas. In the experiments, however, the inconsistent initial temperatures coupled with unaccounted variables, such as radiated body heat, lead to much larger temperature increases. Although there were numerical differences, the trend of the largest temperature increase occurring directly over the tumor remained consistent.

## 6. Discussion

The simulation and experimental results were consistent in detecting abnormal breast tissue through the increased surface temperature of the breast model after electromagnetic excitation. However, the breast phantom used in the experiment was nearly completely homogeneous. Because of the heterogeneity of the tissues within an actual breast, and the diversity in the tissue distribution between different individuals, accurately producing the same results in a clinal setting would be a challenge. As previously mentioned, the mechanics of heat transfer between tissues have not been well described. In addition to that, the different tissues within the breast are not isolated from one another, but amalgamate to form its structure. The blood vessels in breasts also have a random distribution and regulate body temperature. This could potentially lead to inaccurate detection results as the blood network could act as a heat sink and disperse excess heat generated from electromagnetic excitation away from the nonhealthy tissue. However, in an actual breast, the unhealthy breast tissue would develop more blood vessels to sustain its increased metabolism. After electromagnetic excitation, the combination of cancerous tissues having a larger temperature increases due to difference in dielectric properties, increased metabolism, and increased blood flow to the area; it is believed that the temperature difference between healthy and nonhealthy tissues will be detectable from the surface of the breast.

## 7. Conclusion

In this paper, a novel breast cancer detection technique was proposed based on conventional thermal imaging in combination with high-frequency excitation and surface temperature analysis. The structure of the human breast was accurately modeled using the high-frequency FEA approach. Subsequently, the proposed method was described, and the detection system was modeled and proved to be effective. Consequently, a testbed was developed so that the simulation results and the feasibility of the detection method could be experimentally verified. The sensitivity and robustness of this detection method in terms of excitation time, tumor size, and tumor location have also been studied and will be published in the future.

## Figures and Tables

**Table 1:** Antenna parameters.

Width	Length	Substrate height	Substrate permittivity	Resonant frequency
14 mm	20 mm	2 mm	20	2.4 GHz

**Table 2:** Electromagnetic and thermal properties of tissues.

	Relative permittivity at 2.4 GHz	Elec. conductivity at 2.4 GHz (S/m)	Density (kg/m^3^)	Specific heat (J/kg/K)	Thermal conductivity (W/m/K)
Skin	38.1	1.44	1109	3390.5	0.37
Breast fat	5.16	0.133	911	2348	0.21
Breast gland	57.3	1.93	1040	2960	0.33
Muscle	52.8	1.71	1090	3421	0.49
Tumor	47	1.46	1050	3852	0.48

## Data Availability

The electromagnetic and thermal data used to support the findings of this study are included within the article.

## Supplementary Materials

Supplementary Materials Appendix Table 1: equipment used for the experiment.
