# Supplementary material for: High-Frequency Excitation and Surface Temperature Analysis of Breast Tissue for Detection of Anomaly
Source: Biomed Res Int. 2023 Feb 8;2023:4406235. doi: 10.1155/2023/4406235 (PMC9935923; doi:10.1155/2023/4406235)
Supplement: Supplementary Materials — Appendix Table 1: equipment used for the experiment. [file 4406235.f1.docx]

SUPPLEMENTAY MATERIAL

Appendix for Technical Information of Equipment Used in Experiments

During experimental testing, a high frequency signal generator, a DC power supply, a spectrum analyzer, two power amplifiers, three power dividers, a vector network analyzer, several thermistors, several patch antennas and a digital thermometer were used to excite the breast and determine the resulting temperature increase. Table 1 provides information about the equipment.

Appendix Table 1 Equipment Used for Experiment

| **Machine** | **Manufacturer** | **Frequency Range** | **Resolution** |
| --- | --- | --- | --- |
| Analog Signal Generator | Keysight | 9 kHz – 3 GHz | 0.001 Hz |
| Spectrum Analyzer | Rohde & Schwarz | 9 kHz – 3 GHz | 1x10^−6^ |
| Vector Network Analyzer | Keysight | 9 kHz – 4.5 GHz | 1 Hz |
| Power Amplifiers | RF Lambda | 0.7 – 3 GHz |  |
| Power Dividers | Instock | 0.689 – 2.7 GHz |  |
| Antennas | Toaglas | Operated at 2.4 GHz |  |
| DC Power Supply | BK Precision |  | 0.01% + 3mV |
| Digital Thermometer | Omega |  | 0.01℃. |
